# Parents’ Level of Knowledge, Attitudes, and Practices and Associated Sociodemographic Factors Regarding Dengue Infection in Children

**DOI:** 10.7759/cureus.48357

**Published:** 2023-11-06

**Authors:** Shaniza Haniff, Ashwin Shive Gowda, Manoj Sharma, Carole Baskin

**Affiliations:** 1 Internal Medicine, University at Buffalo, New York, USA; 2 Neurology, Royal Stoke University Hospital, Stoke-on-Trent, GBR; 3 Clinical Sciences, St. George's University School of Medicine, St. George, GRD; 4 Health Protection: Preventing and Managing Communicable Diseases, University of Liverpool, Liverpool, GBR

**Keywords:** primary dengue infection, caribbean science and public health, parent survey, knowledge-attitude-practice, dengue virus infection

## Abstract

Background

Dengue infection (DI) continues to rise in the Caribbean. Children are primarily affected by severe infection in this region. Parents thus play an essential role in identifying symptoms, seeking medical care, and preventing DI in their children. Grenada has been endemic to DI since 1956, and to date, no study has assessed the level of knowledge, attitude, and practices (KAP) among parents regarding DI.

Objective

To determine the level of parents' KAP and associated sociodemographic factors regarding DI in children in Grenada.

Methods

A quantitative cross-sectional study of 360 randomly selected parents attending postnatal and child-health clinics was conducted in Grenada. Parents completed a validated survey on demographic information and KAP regarding DI from September to December 2019. KAP scores were calculated and categorized as adequate or inadequate based on the percentage of correctly answered questions on the survey. Chi-square and logistic regression analyses were used to determine the level of KAP and associated sociodemographic factors among parents.

Results

Only 33.9% of parents had adequate knowledge regarding DI, which was independently associated with gender (p-value: 0.001, CI: 1.688-7.411), age (p-value: 0.001, CI: 0.037-0.443), occupation, education, and marital status. Nevertheless, most parents had a positive attitude toward DI prevention (56.4%) and practiced adequate prevention (73%) against DI. Higher education was independently associated with a positive attitude toward DI prevention (p-value: 0.013, CI: 0.190-0.825). However, no sociodemographic factors were associated with the prevention practices of DI.

Conclusion

This study revealed a low level of knowledge among parents, despite an adequate attitude and level of prevention against DI in Grenada. It identified specific groups, such as young, single parents of low educational status, as potential targets for educational campaigns to reduce DI-associated morbidity and mortality among Grenadian children.

## Introduction

Dengue infection (DI) is the fastest-growing mosquito-borne viral infection in the world, transmitted by Aedes aegypti and Aedes albopictus mosquitoes [1]. It is estimated that half the world’s population is at risk of DI, resulting in 400 million infections annually [1]. DI is characterized by a high fever with two or more of the following symptoms: headache, muscle and joint pain, vomiting, and rash in an endemic region. Unlike adults, children with DI may present nonspecific symptoms such as cough, vomiting, and diarrhea [2]. These symptoms can mimic many viral infections, posing a diagnostic challenge [3]. Children are also more susceptible to bleeding and shock, which can facilitate the progression to severe DI [2]. Unsurprisingly, severe DI is a leading cause of death in children in endemic countries, including the Caribbean [1].

DI has been endemic in the Caribbean since the 17th century and provides a favorable rainy climate for the Aedes mosquito to breed, resulting in periodic outbreaks [4,5]. The tri-island state of Grenada, with an estimated annual rainfall of 150 cm (59 inches) in coastal lands, reported its first case of DI in 1956 [5]. Despite over a million cases of DI and hundreds of deaths resulting from it in the Caribbean, DI has only gained significant public health attention in the last few decades due to the increasing number of outbreaks and case-fatality rates associated with severe DI [1,5]. The highest incidence of severe DI occurs in children under 15 years of age in the Caribbean [6]. In recent years, confirmed cases in Grenada have continued to rise, with 151, 239, and 463 cases in 2016, 2017, and 2018, respectively. The neighboring island of Jamaica confirmed an outbreak of 986 cases of DI in 2018, which was 4.5 times the number reported in 2017 [1]. Consequently, in January 2019, the Caribbean Public Health Agency (CARPHA) urged the region to prepare for a severe DI outbreak. While all age groups in the Caribbean were affected, in 2001, 71% of severe DI cases were among children under 15 years [5].

Grenada’s Ministry of Health (MOH) has implemented health education and vector control campaigns to prevent an imminent outbreak of DI. Since there is no specific treatment for DI, the MOH urges all individuals to participate in preventative practices and parents to be vigilant for signs and symptoms of DI in children. If severe DI in children is suspected, indicated by symptoms such as abdominal pain and/or bleeding in stools, early detection and access to medical care within 24-48 hours can reduce the mortality rate from 40-50% to less than 1% [1]. Thus, parental knowledge of the signs and symptoms can promote a positive attitude toward health-seeking behaviors for their children and motivate them to prevent DI [7]. This research utilizes a cross-sectional survey in Grenada to assess the knowledge, attitudes, and practices (KAP) and associated sociodemographic (SD) factors concerning DI in children. To date, no such research has been conducted in Grenada, and therefore, this study aims to fill this gap.

## Materials and methods

Study design

A quantitative cross-sectional study was conducted from September to December 2019 to assess the level of parents’ KAP and associated SD factors regarding DI in children in Grenada. The chosen time period correlates with the rainy season in Grenada and hence increased prevalence of DI [5].

Setting

**Figure 1:**
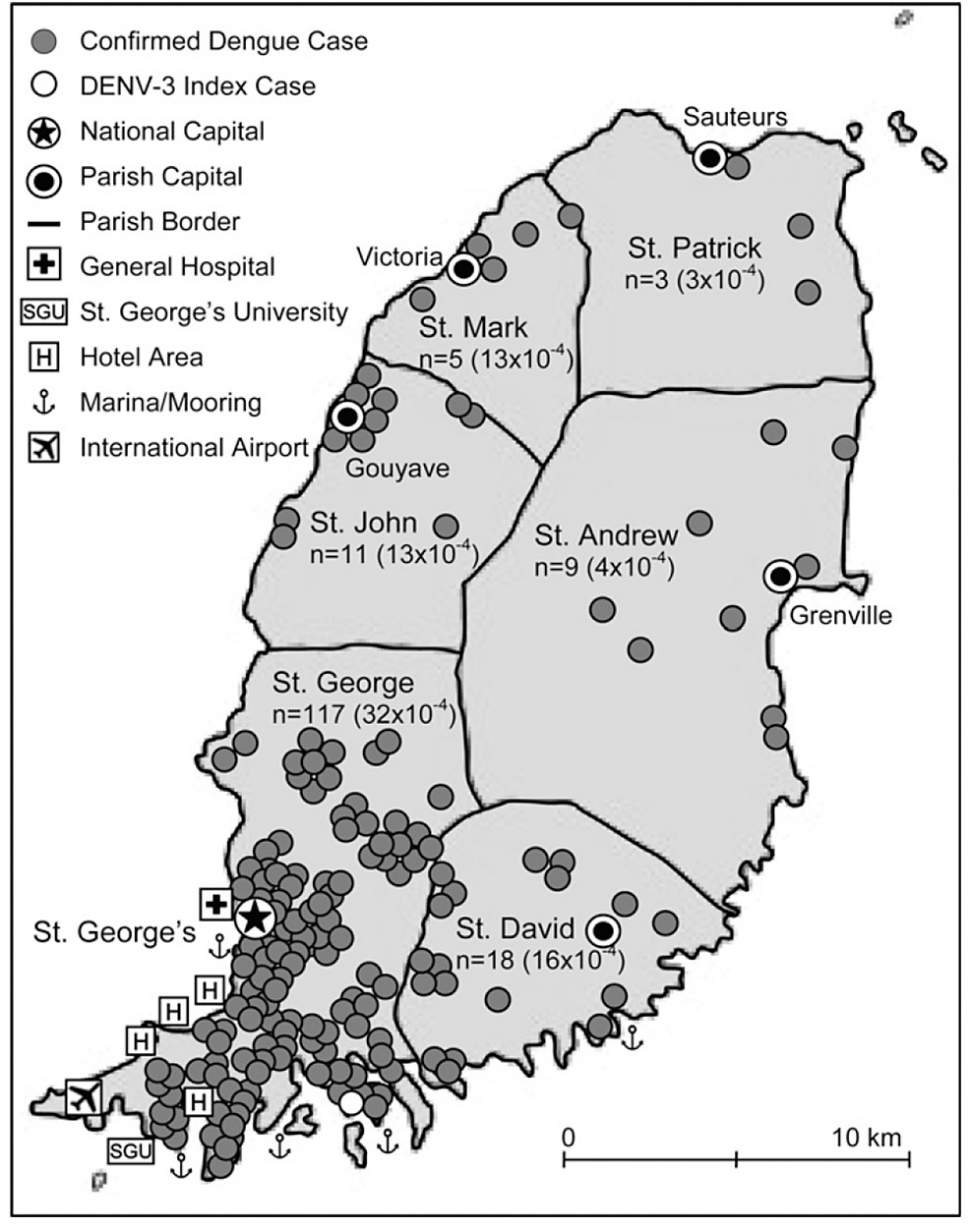
Spatial Distribution of Confirmed Dengue Cases From October 2001 to June 2002 Reproduced with permission from Schiøler K, Macpherson C: Spatial distribution of confirmed dengue cases on the island of Grenada. Am J Trop Med. 2009, 81:283. 10.4269/ajtmh.2009.81.280

The tri-island state of Grenada, located north of Trinidad and Tobago and Venezuela, comprises Grenada, Carriacou, and Petite Martinique. The state covers 440 square kilometers (~109,000 acres) and has a population of 108,000. It is divided into six parishes. This study was conducted in clinics (postnatal and child health) in three of Grenada's parishes: Saint George, Saint David, and Saint Andrew. Saint George, located in the southwest, has the largest population (~37,057), followed by the largest parish, Saint Andrew (~24,749). Saint George, Saint David, and Saint Andrew represent the island's urban, peri-urban, and rural regions, respectively [5]. Together, the three parishes have a substantial landmass, population, and prevalence of DI, making them representative of Grenada (Figure 1). The early French, British, and other European invasions have influenced Grenadian culture, which later adopted a West Indian lifestyle. Once ruled by the British, the island remains an English-speaking nation and adheres to the British political and educational systems. About 75% of the population is of African descent, followed by those of East Indian and European descent [8]. Christianity is the predominant religion, with Islam also practiced.

Sampling approach

Sampling Frame

The sampling frame comprises all 24 healthcare facilities within the three parishes. All hospitals, health centers, and five randomly selected medical stations were included in this study. A total of 10 healthcare facilities were included: Saint George (one hospital, one health center, one medical center), Saint David (one hospital, one health center, two medical stations), and Saint Andrew (one health center, two medical stations).

The population comprised all parents meeting the inclusion and exclusion criteria who attended postnatal and child-health clinics of the selected healthcare facilities.

The study population included parents who attended the postnatal and child-health clinics at selected healthcare facilities within the parishes of Saint George, Saint David, and Saint Andrew in Grenada: (1) two hospitals, one in Saint George and one in Saint Andrew. All parishes refer patients in need of advanced medical care to hospitals. Thus, hospitals contain a diverse population in Grenada. (2) Three health centers, one per parish. Health centers represent primary healthcare in Grenada. (3) Five randomly selected medical stations out of 19, one in Saint George, two in Saint David, and two in Saint Andrew. Medical stations represent the first point of care with healthcare in Grenada. 

It also included parents of at least one child less than 15 attending the selected healthcare facilities' postnatal or child-health clinic. Clinics provide easy and quick access to parents of children less than 15 years of age, which represents the age group primarily affected by severe DI in the Caribbean. In addition, parents over 18 years of age and can speak English were included. Parents under 18 years of age were ethically limited to participate, and surveys were printed in English. Parents over 18 years old who spoke English and were illiterate were assisted in filling out the survey by the researcher.

The exclusion criteria included the following: (1) health facilities that were not operational at the time of the study; (2) health facilities that did not conduct postnatal or child-health clinics; and (3) caregivers (any person other than a biological parent or adoptive) of children less than 15 years of age.

Sample Size

A sample of 360 participants was used in this study. Calculations were made using OpenEpi with the following parameters: Grenada’s population of 108,000, 32% anticipated frequency of adequate knowledge [9], confidence limit ±5, and a design effect of 1. The sample size was adjusted for a non-response rate of 6% in the pilot study to yield a final sample size of 360. The power of assessing the association between the KAP scores and SD factors regarding DI was 83% using IBM SPSS Statistics for Windows, Version 25 (Released 2017; IBM Corp., Armonk, New York).

Recruitment

The first 36 parents attending postnatal and child-health clinics were recruited on presentation at the health facility (n=360). The clinic receptionist distributed recruitment flyers and participant information sheets as parents were registered to reduce coercion [10]. Parents who wished to participate were provided a consent form and survey to complete in a private room in the clinic and subsequently entered a sealed collection box. The researcher read the surveys to participants who were illiterate and assisted some participants in filling out the survey while they nursed their infants.

Data collection methods

Instrument

A validated survey was adapted from: “Knowledge, attitudes, and practices regarding DI in Westmoreland, Jamaica.” Three Delphi review rounds verified the instrument’s purpose, validity, and reliability in the parent study [4]. This research instrument was chosen because of its robustness, similar purpose, and research context in the Caribbean.

The quantitative survey comprised 50 questions in four sections. Section (1) included five demographic questions on gender, age, education, marital status, and occupation. Section (2) consisted of 29 questions regarding the knowledge of DI: six on the signs and symptoms, seventeen on the transmission, and five on the management. Section (3) has three questions on attitude toward DI prevention, and Section (4) has 13 questions: 12 on prevention against DI and one on the source of information on DI. Participants took approximately 30 to 45 minutes to complete the survey.

Pilot Study

A pilot study was conducted in Grenada to assess the survey for comprehensiveness, wording, validity, and reliability. Based on the results of the pilot study, two demographic questions were revised, "education" and "occupation," to replace colloquial terms suited for the Grenadian population. Education was categorized as less than primary, primary, secondary, tertiary, and/or post-graduation, and occupational status was categorized as employed or unemployed. Similarly, three knowledge-based questions were revised to include features of DI specific to children: (1) Is vomiting a sign of dengue fever? (2) Is bleeding from the gums a sign of severe dengue? (3) Is passing blood with stools a sign of severe dengue?

Ethical Considerations

Ethical approval of the research was obtained from the International Review Boards (IRB) of Windward Islands Research and Education Foundation (WINDREF) in Grenada and the University of Liverpool in the United Kingdom. Grenada’s Ministry of Health provided clearance to research in all healthcare facilities following IRB approval.

Analytical approach

A coding frame was developed for each question in the survey. This process was essential to transfer the data from the survey into a numeric form for analysis using SPSS version 25. Gender, marital status, and occupation were listed as nominal data; age as a continuous variable; and education as an ordinal variable [11]. Correct answers for KAP questions were coded “1,” and incorrect answers “0.” The KAP scores were labeled scaled data because of their numeric value. The highest KAP scores in each section were 29 for knowledge, 3 for attitude, and 12 for preventive practice.

Participants who correctly answered 70% or more of the questions on knowledge were considered as having adequate knowledge regarding DI, and those who answered less than 70% of the questions on knowledge were regarded as having inadequate knowledge regarding DI [4]. Similarly, participants who correctly answered 70% or more of the questions on attitude were considered to have an adequate/positive attitude toward DI prevention, and those who answered less than 70% correctly were considered to have an inadequate attitude toward DI prevention. Prevention scores were assessed similarly and recoded into adequate or inadequate prevention against DI.

Frequency tables, histograms, and scatterplots were used to describe the data, determine the distribution, and identify outliers. The analysis did not include missing survey data, ensuring unbiased and reliable results. The chi-squared test was used to identify any significant difference between the dependent variable (level of KAP) and the independent variables (SD factors such as gender, marital status, and occupational status).

Bivariate logistic regression was performed to measure the strength of any association between the level of KAP and each SD factor using crude odds ratio (OR), 95% confidence interval (CI), and a p-value of <0.05 to determine statistical significance. Each significant result from the bivariate analysis was entered into a multivariate regression model to identify any confounding factors (such as gender, age, education level, marital status, and occupation). If an association between the level of KAP and SD factors was significant in the bivariate analysis but not in the multivariate analysis, then the association between the variables was likely due to a confounding factor [12]. It would, therefore, be concluded that there was no independent association between the level of KAP and the SD factor. Statistically significant associations from the multivariate analysis were thus interpreted as independent associations between the level of KAP and the SD factors.

## Results

SD factors among participants

Most participants were females (88.6%), and 89% were between 20 and 39 years of age (Table 1). A minority of participants were under 20 (6.1%, n=22) and above 39 years of age (5%). Many participants' highest level of education was secondary (50.8%, n=183), followed by tertiary and postgraduate studies (31.9%). Together, 17.2% of participants had primary education or less. There was a fairly even representation of marital status and occupation among participants: single, divorced, or widowed (59.4%) versus married or in a common-law union (40.6%), and unemployed (46.1%) versus employed (53.9%).

**Table 1:** Sociodemographic Factors Among Participants

Variables	Number (%)
Gender	
Male	41 (11.4)
Female	319 (88.6)
Age (years)	
Less than 20	22 (6.1)
20-24	78 (21.7)
25-29	78 (21.7)
30-34	82 (22.8)
35-39	82 (22.8)
>39	18 (5.0)
Educational status	
Less than primary	9 (2.5)
Primary	53 (14.7)
Secondary	183 (50.8)
Tertiary and‎/or post-graduate	115 (31.9)
Marital status	
Single, divorced, or widowed	214 (59.4)
Married or common-law union	146 (40.6)
Occupation	
Unemployed	166 (46.1)
Employed	194 (53.9)

Knowledge of DI among participants

Knowledge of Signs and Symptoms

More than half the participants knew fever (86.4%), vomiting (65.3%), and headache (73.6%) as symptoms of DI (Table 2). However, participants were less familiar with symptoms of severe DI: bleeding from the gums and passing blood with stool (23.3% and 30.8%, respectively).

Knowledge of Transmission

Most (62.2%, n=224) knew the type of mosquito that transmits the dengue virus: Aedes mosquito. However, participants also incorrectly believed the dengue virus was transmitted by all species of mosquitoes (41.9%), flies (39.7%), blood transfusion (64.7%), and/or sexual intercourse (43.1%). Only 13.6% (n=49) of participants knew the correct biting time of the Aedes mosquito during the day. Regarding knowledge of prevention against DI, many participants were knowledgeable about the use of window screens and bed nets (75.8%), insecticide sprays (78.6%), and mosquito repellents (75.8%) to reduce mosquitoes. Additionally, many felt removing standing water and covering water containers can prevent mosquito breeding (90.6% and 88.9%, respectively).

Knowledge of Management

Regarding the knowledge of DI management, 60% (n=216) of participants correctly stated they would not give their child aspirin, whereas 16% of participants stated they would. More than half the participants would give their child plenty of fluids and rest if DI was suspected. Almost all participants (n=349) had positive health-seeking behavior (HSB) of consulting a doctor if they suspected DI in their child. However, 68.3% (n=246) believed there was a treatment for DI, while only 10% correctly knew there was no specific treatment for DI.

**Table 2:** Knowledge of Dengue Infection Among Participants

Variable	Yes, n (%)		No, n (%)	
Knowledge of signs and symptoms of dengue infection				
Is fever a symptom of dengue?	311 (86.4)		49 (13.6)	
Is vomiting a symptom of dengue?	235 (65.3)		125 (34.7)	
Is rash a symptom of dengue?	179 (49.7)		181 (50.3)	
Is headache a symptom of dengue?	265 (73.6)		95 (26.4)	
Is abdominal pain a symptom of SD?	154 (42.8)		206 (57.2)	
Is bleeding from the gums a sign of SD?	84 (23.3)		276 (76.7)	
Is passing blood with stool a sign of SD?	111 (30.8)		249 (69.2)	
Knowledge of transmission				
Do flies transmit dengue fever?	143 (39.7)		217 (60.3)	
Do ticks transmit dengue fever?	127 (35.3)		233 (64.7)	
Do all types of mosquitoes transmit dengue fever?	151 (41.9)		209 (58.1)	
Does the Aedes mosquito transmit dengue fever?	224 (62.2)		136 (37.8)	
Does person-to-person contact transmit dengue fever?	172 (47.8)		188 (52.2)	
Can dengue fever be transmitted by blood transfusion?	233 (64.7)		127 (35.3)	
Can dengue fever be transmitted by needle stick?	205 (56.9)		155 (43.1)	
Can dengue fever be transmitted by sexual intercourse?	155 (43.1)		205 (56.9)	
When are the dengue mosquitoes likely to feed/bite?	n (%)			
Nighttime	48 (13.3)			
Day time	49 (13.6)			
Both day and night	245 (68.1)			
Don't know	18 (5.0 )			
Variable	Don’t know, n (%)	No, n (%)		Yes, n (%)
Mosquitoes breed in standing water	23 (6.4 )	12 (3.3)		325 (90.3)
Window screens and bed nets reduce mosquitoes	18 (5.0)	69 (19.2)		273 (75.8)
Insecticide sprays reduce mosquitoes	28 (7.8 )	49 (13.6)		283 (78.6)
Covering water containers reduces mosquitoes	14 (3.9)	26 (7.2)		320 (88.9)
Removal of standing water can prevent mosquito breeding	20 (5.6 )	14 (3.9)		326 (90.6)
Mosquito repellants prevent mosquito	30 (8.3)	57 (15.8)		273 (75.8)
Cutting down bushes can reduce mosquitoes	33 (9.2)	32 (8.9)		295 (81.9)
Pouring chemicals in standing water can kill mosquito larvae	47 (13.1)	32 (8.9)		281 (78.1)
Knowledge of management				
Would you give your child an aspirin for dengue infection?	88(24.4)	216 (60.0)		56 (15.6)
Would you give your child plenty of rest for dengue infection?	45(12.5)	52 (14.4)		263(73.1)
Would you give your child plenty of water for dengue infection?	30 (8.3)	13 (3.6)		317(88.1)
Would you consult a doctor if your child has dengue infection?	n (%)			
No	5 (1.4)			
Yes	349 (96.9)			
Is there a treatment for dengue infection?	n (%)			
Don’t know	78 (21.7)			
No	36 (10.0)			
Yes	246 (68.3)			

Attitude toward DI prevention among participants

Most participants (83.9%) identified DI as a serious illness (108 agreed, 194 strongly agreed). Many (n=236) believed their child was at risk of being infected with dengue virus, whereas 34.4% did not share this view. Nevertheless, 85.3% of participants believed DI could be prevented (53.1% strongly agreed, 32.2% agreed).

Prevention against DI among participants

Participants stated they engaged in preventive practices against DI by using bed nets (84.2%), insecticide sprays (88.1%), and window screens (81.1%). Less common preventive practices against DI included the use of mosquito-eating fishes (42.5%, n=156). Covering water containers at home, and cleaning water containers and drains around the house was readily (often and always) done by 86% of participants. The KAP among participants regarding DI was influenced by the television or radio (46.7%) and health workers (34.4%).

Level of KAP regarding DI 

**Figure 2:**
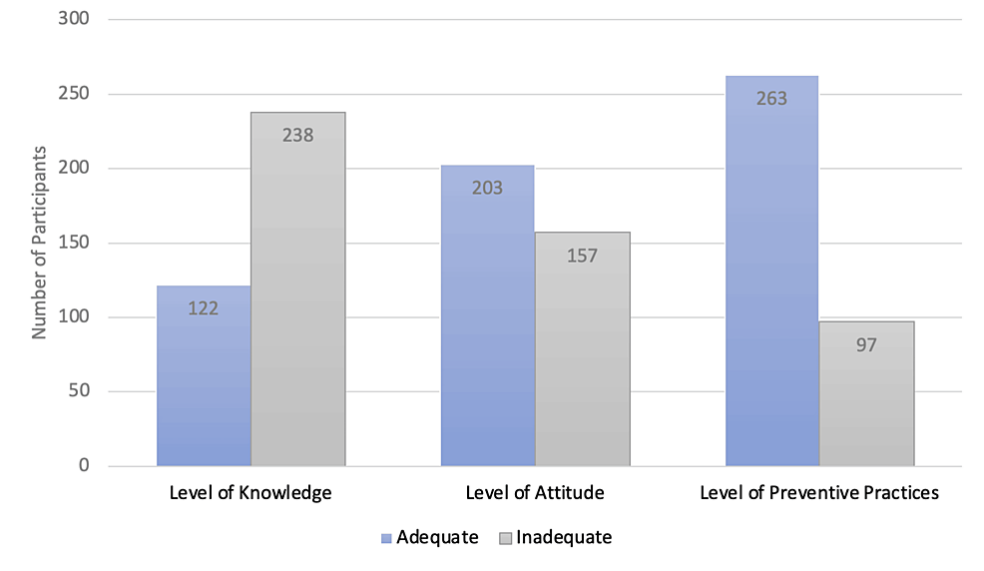
Level of Knowledge, Attitude, and Practices Regarding Dengue Infection Among Participants

Only 33.9% (n=122) of participants answered at least 70% of the knowledge questions correctly and thus were determined to have an adequate level of knowledge regarding DI (Figure 2). However, 56.4 % (n=238) of participants answered more than 70% of the questions on attitude correctly, constituting an adequate/ positive attitude toward DI prevention. Similarly, 73% (n=263) of participants answered more than 70% of the questions on prevention correctly and thus were considered to have an adequate level of prevention against DI.

The association between the level of knowledge of DI and SD factors 

All SD factors were statistically associated with adequate knowledge regarding DI in the bivariate analysis (Table 3). Males were more likely to have adequate knowledge regarding DI compared to females (p-value: 0.015, CI: 1.176-4.368). After adjusting for potential confounding factors, the multivariate analysis showed males were 3.5 times more likely to have an adequate level of knowledge regarding DI compared to females (p-value: 0.001, CI: 1.688-7.411).

**Table 3:** The Association Between the Level of Knowledge of Dengue Infection and Sociodemographic Factors Among Participants OR, odds ratio; CI, confidence interval The level of significance used is p < 0.05

Variable	Crude Sig.	Values OR (95% C.I.)	Adjusted Sig.	Values OR (95% CI)
Gender	.015		.001	
Male	.015	2.266 (1.176-4.368)	.001	3.537 (1.688-7.411)
Female		Referent		
Age (years)	0.001		.0030	
Less than 20	.038	.222 (.054-.923)	.057	224 (.048-1.047)
20-24	.228	.529 (.188-1.490)	.128	.401 (.124-1.301)
25-29	.004	.200 (.067-.600)	.001	.127 (.037-.443)
30-34	.342	.608 (.218-1.696)	.165	.441 (.139-1.401)
35-39	.778	.864 (.311-2.397)	.432	.633 (.202-1.981)
More than 39		Referent		
Educational status	.017		.004	
Less than primary	.198	.346 (.069-1.738)	.135	.257 (.043-1.527)
Primary	.012	.394 (.191-.813)	.001	.236 (.103-.539)
Secondary	.008	.521 (.321-.845)	.023	.540 (.317-.919)
Tertiary and‎/or post-graduate		Referent		
Marital status	.001		.035	
Single or divorce or widowed	.001	.476 (.305-.743)	.035	.576 (.345-.963)
Married or common-law union		Referent		
Occupation	.003		.392	
Unemployed	.003	.509 (.324-.798)	.392	799 (.478-1.336)
Employed		Referent		

Younger age was statistically associated with lower odds of having adequate knowledge of DI in the bivariate analysis (p-value: 0.001). Participants under 20 years of age and those between the ages of 25 and 29 were less likely to have an adequate level of knowledge regarding DI compared to participants over 39 years of age. However, after adjusting for confounding factors (gender, occupation, educational status, and marital status) in the multivariate analysis, only the participants between the ages of 25 and 29 were less likely to have an adequate level of knowledge regarding DI compared to those older than 39 years of age. The multivariate analysis showed these participants (ages 25-29) were 87% less likely to have adequate knowledge regarding DI compared to participants over 39 years of age (p-value: 0.001, CI: 0.037-0.443).

A lower level of education (primary and secondary) was associated with lower odds of having adequate knowledge regarding DI compared to those who had completed tertiary and/or post-graduate studies in the bivariate analysis (p-value: 0.017). Participants with only primary education were 76% less likely (p-value: 0.001, CI: 0.103-0.539), and those with only secondary education were 50% less likely (p-value: 0.023, CI: 0.317-0.919) to have an adequate level of knowledge regarding DI compared to those who had completed tertiary and/or post-graduate studies. Therefore, education was independently associated with adequate knowledge regarding DI.

Being single, divorced, or widowed was associated with lower odds of having adequate knowledge regarding DI compared to being married or in a common-law union (p-value: 0.001, CI: 0.305-0.743). In the multivariate analysis, participants who were single, divorced, or widowed were 42% less likely to have an adequate level of knowledge regarding DI compared to those who were married or in a common-law union (p-value: 0.035, CI: 0.345-0.963). Similarly, unemployed participants were less likely to have adequate knowledge regarding DI compared to those who were employed (p-value: 0.003, CI: 0.324-0.798). However, this association was not independently associated with adequate knowledge regarding DI in the multivariate analysis (p-value: 0.329, CI: 0.478-1.336). Thus, the relationship between employment and level of knowledge regarding DI was likely due to confounding variables.

The association between attitude toward DI prevention and SD factors 

Only education was associated with a positive attitude toward DI prevention in the bivariant and multivariate analyses (Table 4). Participants with only a primary level of education were 61% less likely (p-value: 0.013, CI: 0.190-0.825), and those with only a secondary level of education were 55% less likely to have a positive attitude towards DI prevention than participants who completed tertiary and/or post-graduate studies.

**Table 4:** The Association Between Attitude Toward Dengue Infection Prevention and Sociodemographic Factors Among Participants OR, odds ratio; CI, confidence interval The level of significance used is p < 0.05

Variable	Crude Sig.	Values OR (95% CI)	Adjusted Sig.	Values OR (95% CI)
Gender	.197		.073	
Male	.197	1.567 (.792-3.100)	.073	1.919 (.940-3.917)
Female		Referent		
Age (years)	.371		.539	
Less than 20	.822	.865 (.246-3.050)	.929	.942 (.254-3.500)
20-24	.361	1.618 (.576-4.539)	.438	1.544 (.515-4.631)
25-29	.535	1.385 (.494-3.882)	.632	1.307 (.437-3.910)
30-34	.171	2.056 (.733-5.768)	.217	1.973 (.670-5.806)
35-39	.203	1.953 (.697-5.472)	.228	1.938 (.662-5.673)
More than 39		Referent		
Educational status	.005		.009	
Less than primary	.856	.875 (.207-3.699)	.972	1.027 (.229-4.605)
Primary	.011	.421 (.216-.823)	.013	.395 (.190-.825)
Secondary	.001	.433 (.265-.708)	.002	.453 (.272-.754)
Tertiary and‎/or post-graduate		Referent		
Marital status	.312		.623	
Single, divorced, or widowed	.312	.803 (.524-1.229)	.623	.887 (.551-1.429)
Married or common-law union		Referent		
Occupation	.232		.979	
Unemployed	.232	.775 (.510-1.177)	.979	1.006 (.634-1.597)
Employed		Referent		

The association between the level of prevention against DI and SD factors 

Education (p-value: 0.036) and occupation (p-value: 0.015) were associated with an adequate level of prevention against DI. However, after adjusting for confounding factors, there was no independent association between SD factors and prevention against DI (p-value > 0.05).

Summary of key findings

The results showed that most participants were females between the ages of 20 and 39 years with secondary education or higher. More than half the participants were familiar with common symptoms of DI, including fever and headaches, but were less familiar with specific signs of severe DI, including bleeding from the gums and passing blood with stool. While more than half the participants were also familiar with the mode of dengue virus transmission by the Aedes mosquito, many also incorrectly believed flies, blood transfusion, and/or sexual intercourse aided in the transmission. Unsurprisingly, only 33.9% of participants had adequate knowledge regarding DI. Despite a low level of knowledge, many had a positive/adequate attitude toward DI prevention, and 70% were adequately engaged in prevention against DI.

The multivariate analysis showed that gender, age, education, and marital status were independently associated with an adequate level of knowledge regarding DI among parents. However, only education was independently associated with a positive attitude toward DI prevention, and there was no independent association between SD factors and the prevention of DI.

## Discussion

This study found that 33.9% of parents had adequate knowledge regarding DI in children in Grenada. This is comparable to similar cross-sectional KAP studies in Jamaica: 13% in 2005 and 54.5% in 2010 [5,13]. This may be because of a similar literacy level, cultural values, and lifestyle practices. Parents in this study were familiar with fever as a symptom of DI, similar to DI studies in Grenada and Colombia conducted in 2005 and 2018, respectively [9,14]. Fever, however, is non-specific and may not be an essential cue for parents to seek medical attention for their child [2]. Parents were also aware of vomiting and headache symptoms associated with DI, similar to a cross-sectional study in Colombia [15], unlike most studies in the Caribbean. This may be because this study was conducted during the peak of an outbreak. Thus, parents may have been sensitized to the information, unlike during other studies in the Caribbean [4,9]. Parents were, however, unfamiliar with symptoms of severe DI in children, including abdominal pain, bleeding from the gums, and passing blood with stool, as seen in many KAP studies among parents [2,16,17]. These symptoms are used to distinguish DI from other infections [18], and parents who recognize these symptoms are more likely to associate it with the severity of the disease, as demonstrated in a cross-sectional study among Malaysian parents [2]. 

The knowledge of dengue virus (DENV) transmission among parents was inconclusive in this study. While more than half of the parents correctly knew the Aedes mosquito transmitted DENV, many also incorrectly believed it was transmitted by blood transfusion and sexual intercourse. This highlights a deficit in the knowledge of the transmission of DENV. However, parents knew bed nets, insecticide sprays, and mosquito repellants prevented mosquito bites and DI. Most parents also correctly stated they would not give their child aspirin if DI is suspected. However, the remaining 40% were unsure or would give their child aspirin. This is a common alarming finding in the Caribbean, as aspirin can exacerbate DI in children, thus highlighting a need to be addressed [4]. Many parents incorrectly believed there was a treatment for DI, which can explain this study's high level of positive health-seeking behavior. This knowledge gap must be addressed since there is no specific treatment for DI, making prevention key. Although parents knew DI was a serious illness that could be prevented, 34.4% did not agree that their child was at risk of infection with DENV. This deficit in the awareness of an imminent outbreak of DI in Grenada among parents is alarming, as cross-sectional studies regarding DI in Venezuela and Indonesia found individuals who felt at risk were more likely to engage in preventive measures [19,20]. Gender, age, education, and marital status were SD factors associated with an adequate knowledge of DI in this study among parents. Male parents were more likely to have adequate knowledge regarding DI than females, unlike other cross-sectional studies in Malaysia, Jamaica, and Colombia [14,21,22]. Males were underrepresented in this study (11.4%); thus, the data may not represent gender distribution in Grenada.

Nevertheless, males were more likely to work outside the home than females, with an increased likelihood of interacting with people who may be knowledgeable about DI. Older parents (more than 39 years of age) and those with a high level of education were found to be more knowledgeable about DI. Older parents were more likely to complete tertiary and post-graduate studies, be involved in the workforce, and be sensitized to DI symptoms and transmission than young parents. The association between the level of knowledge and education is common in cross-sectional KAP studies regarding DI [13,14]. Finally, married parents were more knowledgeable than those who were single. In Grenada, single parents are more common than married parents, as captured in this study (59.4% vs. 40.6%, respectively). Single parents have a heavy responsibility and may be less likely to engage in health education campaigns regarding DI than married parents. Grenada also has a large young population: 57% under the age of 35 years. Single parents, especially of younger generations, may be less likely to complete school and, therefore, readily understand the information presented to the public on DI. Thus, the low level of knowledge regarding DI in Grenada among parents in this study may result from many young single parents who have completed a secondary level of education or less.

More than half of the parents in this study had a positive attitude toward DI prevention and HSB. This mirrors a cross-sectional study conducted in India in 2018 [16] and may result from a similar methodology: surveying a healthcare setting among parents. Participants present in a healthcare setting may already be inclined to have a positive attitude towards DI prevention and HSB. In support of this, contrasting results were seen in a cross-sectional study in Jamaica conducted outside of a healthcare setting, where less than half the participants had a positive attitude toward preventing DI [4]. Another cross-sectional study found that households with children were more likely to have a positive attitude toward DI prevention than those without children [13]. Therefore, parents may be inherently protective against any illness in their children [23] and have an intrinsic positive attitude towards disease prevention and HSB. This may explain why, despite a low level of knowledge among parents in this study, a positive attitude and adequate prevention against DI were seen.

This study also showed parents with a high level of education were more likely to have a positive attitude toward DI prevention. While most parents were also actively engaged in preventive practices against DI. Interestingly, SD factors were not associated with preventing DI in this study. This contrasts with a cross-sectional study conducted in Malaysia, where gender and age were associated with preventing DI [20]. The difference in findings may be because, in Grenada, parents were routinely involved in hygienic practices such as covering water containers, using window screens, and cutting down bushes in the yard regardless of SD factors. Though not exclusive to the prevention of DI, these standard practices may be practiced for hygiene and prevention of infectious illnesses and thus unknowingly offer protection against DI.

Reliability, validity, and generalizability

This study was conducted among a large population with a high DI prevalence. Using a random sampling method, objective data collection with a validated research instrument, appropriate statistical analysis, and adjustment for confounders during data analysis increased the internal and external validity. Furthermore, the baseline SD factors of participants mirror that of the Grenadian parent population, and the targeted sample size was met to ensure adequate power and identification of associations between the level of KAP and SD factors of DI. Therefore, this study does have generalizability to the Grenadian parent population. 

Limitations of study

A parent population outside a healthcare setting would provide a better representative and unbiased study population. Additionally, an equal representation of male and female parents may provide more information regarding the association between gender and the level of KAP regarding DI among parents. The researcher also assisted some participants who were illiterate in filling out the survey, which could contribute to observation bias. Therefore, the study results should be interpreted in light of the limitations mentioned.

Recommendations

Health education campaigns have been the mainstay of increasing awareness of DI in Grenada [9]. A successful health education campaign adapts critical messages for its target population. Therefore, the deficit in parents' knowledge regarding DI in Grenada should be addressed using existing health education campaigns with adapted vital messages. These key messages should include signs and symptoms specific to DI in children and those more indicative of severe DI, such as bleeding from gums and passing blood with stool. Common misconceptions, such as the spread of the dengue virus through flies, ticks, needle sticks, and sexual intercourse, should also be addressed to ensure correct prevention against DI. Parents should also be aware of the importance of preventing DI since there is no treatment for DI.

It is equally important to convey these key messages across reliable and accessible media. Parents in this study stated that the media (television and radio) and healthcare workers played an essential role in their knowledge of DI. Therefore, these avenues can be utilized to increase the level of knowledge among parents regarding DI in children in Grenada [24]. However, these campaigns must also be implemented with consideration for young single parents with low educational status [25]. This is important since the severe DI factors mentioned above were associated with a low level of knowledge regarding DI in this study. A cross-sectional study in Vietnam demonstrated a successful increase in the level of KAP regarding DI among mothers after implementing targeted health education campaigns [7]. Specifically, the approach to parents of low educational status included using simple words, slow speech, and repetition of information by healthcare workers. Using pictures, flyers, and less text in the media during health education campaigns can also target parents with low educational status. Therefore, emphasis must be placed on tailored campaigns for different segments of the population, considering their educational level, marital status, and other sociodemographic variables.

Parents’ exposure to health education campaigns alone may not be sustainable in improving behavioral change [26]. This can be complemented by school health education campaigns to foster sustainable change in the prevention of DI among parents [27]. Health education campaigns focused on children can raise the awareness of both parents and teachers, which in turn encourages prevention among households, communities, and public institutions [28]. This leads to an integrated community-based approach to prevent DI and can bridge the knowledge gap, ensuring sustainable positive attitudes and prevention against DI in Grenada. Adopting an integrated approach encourages parents, teachers, children, and all community members to prevent DI, encourage intersectoral relationships, and develop policies for sustainable KAP regarding DI [29].

Lastly, legislation regarding the prevention against DI should be reinforced to ensure sustainability. This includes frequent monitoring and evaluation of the health education campaigns at the level of healthcare, schools, and communities, the documented success of which was seen in Singapore [30]. Implementing these measures would require a multidisciplinary team, including public health officials, the educational sector, the public, and the media [29]. Together, the intersectoral team can promote maximum success for health education campaigns regarding DI and can lead to positive health outcomes.

## Conclusions

This study provides insight into the level of KAP and associated SD factors regarding DI among parents in Grenada. There needs to be more knowledge regarding DI among parents in Grenada, which is associated with low levels of education, younger age, and single marital status despite positive attitudes and prevention against DI. These findings can guide future health education campaigns and interventions to ensure sustainable KAPs against DI among parents in Grenada. It also allows future KAP studies regarding DI in Grenada to understand, assess, and improve the fight against DI.

## References

[1] World Health Organization (2019). Dengue fever-Jamaica. https://www.who.int/csr/don/4-february-2019-dengue-jamaica/en/.

[2] Ariffin F, Ramli AS, Naim N, Selamat MI, Syed-Jamal SJ (2014). Recognizing life-threatening features of dengue in children and health seeking behavior in dengue emergency amongst parents and carers: a cross-sectional study in Gombak District, Malaysia. Med J Malaysia.

[3] Elling R, Henneke P, Hatz C, Hufnagel M (2013). Dengue fever in children: where are we now?. Pediatr Infect Dis J.

[4] Shuaib F, Todd D, Campbell-Stennett D, Ehiri J, Jolly PE (2010). Knowledge, attitudes and practices regarding dengue infection in Westmoreland, Jamaica. West Indian Med J.

[5] Schiøler KL, Macpherson CN (2009). Dengue transmission in the small-island setting: investigations from the Caribbean island of Grenada. Am J Trop Med Hyg.

[6] Istúriz RE, Gubler DJ, Brea del Castillo J (2000). Dengue and dengue hemorrhagic fever in Latin America and the Caribbean. Infect Dis Clin North Am.

[7] Tram TT, Anh NTN, Hung NT (2003). The impact of health education on mother’s knowledge, attitude and practice (KAP) of dengue haemorrhagic fever. Dengue Bulletin.

[8] (2023). About Grenada. http://www.gov.gd/about_grenada.html.

[9] Panagos A, Lacy ER, Gubler DJ, Macpherson CN (2005). Dengue in Grenada. Rev Panam Salud Publica.

[10] Manti S, Licari A (2018). How to obtain informed consent for research. Breathe (Sheff).

[11] Ranganathan P, Gogtay NJ (2019). An introduction to statistics - data types, distributions and summarizing data. Indian J Crit Care Med.

[12] Pourhoseingholi MA, Baghestani AR, Vahedi M (2012). How to control confounding effects by statistical analysis. Gastroenterol Hepatol Bed Bench.

[13] Alobuia WM, Missikpode C, Aung M, Jolly PE (2015). Knowledge, attitude, and practices regarding vector-borne diseases in western Jamaica. Ann Glob Health.

[14] Diaz-Quijano FA, Martínez-Vega RA, Rodriguez-Morales AJ, Rojas-Calero RA, Luna-González ML, Díaz-Quijano RG (2018). Association between the level of education and knowledge, attitudes and practices regarding dengue in the Caribbean region of Colombia. BMC Public Health.

[15] Castro-Bonilla L, Coronel-Ruiz C, Parra-Alvarez S, Castellanos JE, Porras-Ramírez A, Velandia-Romero ML (2018). Factors associated with dengue virus infection and reinfection in asymptomatic children in two Colombian municipalities. Am J Trop Med Hyg.

[16] Harish S, Srinivasa S, Shruthi P (2018). Knowledge, attitude and practice regarding dengue infection among parents of children hospitalized for dengue fever. Int J Contemp Pediatr.

[17] Laghari T, Memon A, Mustufa M (2015). Assessment of mass level public awareness campaigns regarding dengue among parents visiting tertiary care children hospital Karachi, Pakistan. Med Health Sci.

[18] Phuong CX, Nhan NT, Kneen R (2004). Clinical diagnosis and assessment of severity of confirmed dengue infections in Vietnamese children: is the world health organization classification system helpful?. Am J Trop Med Hyg.

[19] Elsinga J, Lizarazo EF, Vincenti MF (2015). Health seeking behaviour and treatment intentions of dengue and fever: a household survey of children and adults in Venezuela. PLoS Negl Trop Dis.

[20] Rakhmani AN, Limpanont Y, Kaewkungwal J, Okanurak K (2018). Factors associated with dengue prevention behaviour in Lowokwaru, Malang, Indonesia: a cross-sectional study. BMC Public Health.

[21] Wong LP, Shakir SM, Atefi N, AbuBakar S (2015). Factors affecting dengue prevention practices: nationwide survey of the Malaysian public. PLoS One.

[22] Lue AM, Richards-Dawson ME, Gordon-Strachan GM (2022). Severity and outcomes of dengue in hospitalized Jamaican children in 2018-2019 during an epidemic surge in the Americas. Front Med (Lausanne).

[23] Hughes P (2010). Paradigms, methods and knowledge. Doing Early Childhood Research.

[24] Amanah MA, Abdullah H, and Abdul Ghafar N (2018). Knowledge attitude and practice on dengue among university students. Int J Community Med Public Health.

[25] Nguyen HV, Than PQ, Nguyen TH (2019). Knowledge, attitude and practice about dengue fever among patients experiencing the 2017 outbreak in Vietnam. Int J Environ Res Public Health.

[26] Parks WJ, Lloyd LS, Nathan MB (2004). International experiences in social mobilization and communication for dengue prevention and control. Dengue Bulletin.

[27] Ooi EE, Goh KT, Gubler DJ (2006). Dengue prevention and 35 years of vector control in Singapore. Emerg Infect Dis.

[28] Kyu HH, Thu M, Van der Putten M (2005). Myanmar migrant woman caretakers on prevention of dengue fever: a study on knowledge, attitude and practices in Tak Province, Thailand. AU J Tech.

[29] Avila Montes GA, Martínez M, Sherman C, Fernández Cerna E (2004). Evaluation of an educational module on dengue and Aedes aegypti for schoolchildren in Honduras (Article in Spanish). Rev Panam Salud Publica.

[30] Kumar S, Preetha G (2012). Health promotion: an effective tool for global health. Indian J Community Med.

